# Lived experiences of social alienation among Chinese women with gynecologic malignancies: a descriptive phenomenological study

**DOI:** 10.3389/fpubh.2026.1842173

**Published:** 2026-07-14

**Authors:** Fengfeng Shen, Lei Ren, Na Zhou, Yiyu Zhuang, Chenping Zhu, Liyan Chen, Aifen Ye

**Affiliations:** Nursing Department, Sir Run Run Shaw Hospital, School of Medicine, Zhejiang University, Hangzhou, Zhejiang, China

**Keywords:** gynecologic malignancy, lived experience, phenomenology, qualitative study, social alienation

## Abstract

**Objective:**

To explore the lived experiences of social alienation among Chinese women with gynecologic malignancies and identify the meanings they attached to social withdrawal and reconnection.

**Methods:**

This descriptive phenomenological study was conducted in a tertiary hospital in Hangzhou, China, in April 2025. Twelve women with histopathologically confirmed gynecologic malignancies were recruited through purposive sampling with maximum variation. Semi-structured face-to-face interviews were audio-recorded, transcribed verbatim, and analyzed using Colaizzi’s seven-step phenomenological method.

**Results:**

Three themes and eight subthemes were identified. Participants described withdrawing from others to protect a threatened identity, including concealing the illness to avoid attention and restricting daily movement and social contact. They also described living in a body that feels changed and exposed, reflected in physical exhaustion and functional limits, changes in appearance and anticipated judgment, and concerns about family reputation and financial strain. Finally, they expressed a desire for understanding and a return to normal life, including feeling misunderstood in relationships and work and seeking work, normality, and meaningful connection. Across accounts, social alienation appeared to arise from the interaction of symptom burden, visible treatment effects, anticipated social judgment, practical constraints, and limited opportunities for understanding and support.

**Conclusion:**

Social alienation among women with gynecologic malignancies is a multidimensional experience shaped by bodily change, social responses, and practical barriers to participation. Supportive care should include psychosocial assessment, family-informed communication, peer support, and individualized assistance with social and occupational reintegration.

## 1 Introduction

Gynecologic malignancies are an important public health concern for women worldwide. According to the World Health Organization (WHO) cervical cancer was the fourth most common cancer among women globally in 2022 (1). It has an estimated 660,000 new cases and 350,000 deaths. In China, cervical cancer remained a major female cancer burden. It ranked fifth among cancers in women with 150,659 new cases in 2022 (1, 2). Although significant advances have been made in surgery, chemotherapy, radiotherapy, and survivorship care many patients continue to experience persistent physical, psychological, and social challenges after diagnosis and treatment (3, 4).

In recent survivorship research more attention has been paid to the social consequences of cancer. These consequences include challenges in relationships, social participation, and sense of belonging. Women with gynecologic malignancies are vulnerable to these difficulties as the treatment often involves fatigue, pain, altered body image. It also causes sexual concerns, fertility-related loss, and disruption of everyday roles (5). These changes may affect how women relate to family members, friends, colleagues, and the wider community. These changes may also contribute to feelings of distance, self-protection, or exclusion from ordinary social life (5, 6).

The present study draws on Goffman’s concept of stigma and spoiled identity (6). It describes how a discrediting attribute may alter a person’s social identity and sense of belonging. In this study, social alienation is understood as a subjective experience of estrangement, disconnection, and reduced belonging in relation to others and the social world (6, 7). It is different from social isolation which refers to limited social contact or participation (7), social alienation reflects how social relationships and interactions are experienced and interpreted. Among women with gynecologic malignancies, treatment-related bodily changes, perceived social judgment, and stigma-related concerns may contribute to emotional distance, social withdrawal, and difficulties in interpersonal relationships (6–10). These experiences may be particularly important within the Chinese sociocultural context, where family reputation and social face (Mianzi) are highly valued (11, 12). Women with gynecologic malignancies are psychosocially vulnerable because the disease and its treatment can influence their reproductive health, sexual wellbeing, and intimate interpersonal relationships (8, 13–16).

Previous studies have reported reduced social support, emotional withdrawal, stigma-related difficulties, and body image concerns among cancer survivors (7–10). Despite growing recognition of the psychosocial challenges associated with gynecologic malignancies, limited research has explored social alienation among these patients. Most of the existing research has relied on quantitative methods, which provide limited understanding of how women perceive and interpret these experiences in their daily lives (8, 12–21). Qualitative research may therefore offer deeper insight into the lived experience of social alienation among women with gynecologic malignancies (11).

Therefore, a descriptive phenomenological approach was adopted to gain deeper insight into how women with gynecologic malignancies experience and interpret social alienation in their daily lives. This study aimed to explore participants’ perceptions of social alienation and factors contributing to these experiences. We also explored their views on social reconnection and participation.

## 2 Methods

### 2.1 Study design and setting

This study used a descriptive phenomenological design to explore the lived experiences of social alienation among women with gynecologic malignancies. The qualitative study formed part of a broader three-phase project that included a cross-sectional survey, the present phenomenological interview study, and a subsequent Delphi study for intervention development. The qualitative phase was designed to deepen understanding of the meanings, perceptions, and needs identified in the first phase. The study was conducted in the gynecologic inpatient ward of a tertiary hospital in Hangzhou, Zhejiang Province, China, in April 2025. We reported the study in accordance with the Consolidated Criteria for Reporting Qualitative Research (COREQ) (22).

### 2.2 Sampling and participant selection

Participants were recruited using purposive sampling with maximum variation to capture a wide range of experiences across age, marital status, education, residence, diagnosis, and disease stage. Inclusion criteria were: (1) histopathologically confirmed gynecologic malignancy; (2) age ≥18 years; (3) awareness of diagnosis; and (4) ability and willingness to provide informed consent and participate in a face-to-face interview. Exclusion criteria were: (1) severe psychiatric illness; (2) cognitive impairment or severe communication difficulties; and (3) serious physical conditions that prevented meaningful participation.

Eligible patients were identified through the ward team and approached by the research team. Participation was voluntary, and refusal had no effect on clinical care. Recruitment continued until data saturation was reached. Saturation was judged pragmatically as the point at which no new codes, meanings, or conceptual insights emerged in consecutive interviews. In this study, saturation appeared to be achieved by the 10th interview. Two additional interviews were then conducted to confirm stability of the thematic structure. The final sample comprised 12 women.

### 2.3 Data collection

#### 2.3.1 Interview guide development

A semi-structured interview guide was developed based on the study objectives and relevant literature. The draft guide was reviewed by the research team and clinicians with expertise in gynecologic oncology and psycho-oncology to ensure clarity, relevance, and cultural appropriateness. The guide was then piloted with two patients to refine the wording and sequence of questions. Pilot interviews were not included in the final analysis. The final interview guide focused on four domains: (1) Changes in life after diagnosis: *“How has your life changed since being diagnosed with a gynecologic malignancy?”;* (2) Social participation: *“How has your participation in social activities changed since diagnosis?”*; (3) Interpersonal relationships: *“What impact has the diagnosis had on your communication and relationships with others?”*; and (4) Social difficulties and support needs: *“What difficulties do you face when interacting with others, and what kind of support would help you most?”*

#### 2.3.2 Interview procedure

Face-to-face interviews were conducted by a female oncology nurse researcher trained in qualitative interviewing. She had no prior therapeutic relationship with the participants. Interviews were conducted in a private consultation room in the hospital at a time convenient for participants. Only the participant and interviewer were present. Interviews were conducted in Mandarin Chinese to allow participants to express themselves in their preferred language. The interviewer began each interview with brief rapport-building and then followed the interview guide flexibly. It allowed participants to elaborate freely on issues important to them. Probing questions were used when necessary to clarify meanings and deepen understanding. All interviews were audio-recorded with participants’ permission and lasted approximately 30–60 min. The interviewer also took field notes during and immediately after each interview to document non-verbal behavior, emotional expression, and contextual observations.

#### 2.3.3 Transcription and translation

Recordings were transcribed verbatim in Chinese by the first author and checked against the audio files by a second researcher for accuracy. Representative quotations were translated into English by the first author and reviewed by a senior bilingual team member to preserve meaning and cultural nuance. All representative quotations were translated into English for publication purposes. Where necessary, culturally specific expressions were retained or briefly explained to preserve meaning.

### 2.4 Data analysis

**Figure 1:**
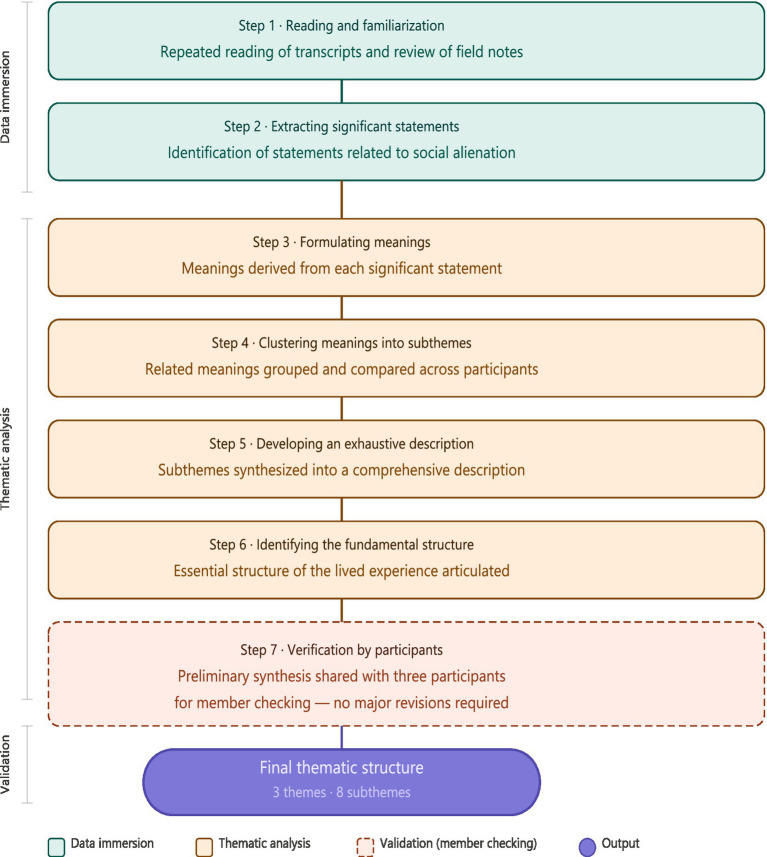
Colaizzi’s seven-step phenomenological analysis process and participant verification procedure used in this study. The figure illustrates the sequential stages of Colaizzi’s phenomenological analysis, including data immersion (Steps 1–2), thematic analysis (Steps 3–6), and validation through participant verification/member checking (Step 7). During the verification stage, a summary of the preliminary themes was shared with three participants to confirm the accuracy and credibility of the findings. No major revisions were required following participant feedback.

The interview data were analyzed using Colaizzi’s seven-step phenomenological method (23) by two researchers independently (FS and LR). The analysis was inductive, meaning that codes and themes were generated from the data rather than imposed *a priori*. The analysis proceeded as follows: (1) Familiarization: both researchers repeatedly read the transcripts while listening to the recordings and reviewing field notes to obtain an overall sense of the data; (2) Extraction of significant statements: statements directly related to social alienation were identified and highlighted; (3) Formulation of meanings: the researchers interpreted each significant statement into a formulated meaning while remaining close to the participant’s account; (4) Theme clustering: related meanings were grouped into subthemes and themes; (5) Exhaustive description: the themes were integrated into a comprehensive description of the phenomenon; (6) Fundamental structure: the essential structure of the lived experience was derived from the thematic description; (7) Verification: As part of Step 7 of Colaizzi’s phenomenological analysis process, a summary of the preliminary themes was shared with three participants to confirm that the findings accurately reflected their experiences. Refer to Figure 1.

The two researchers compared their coding and theme development regularly to enhance analytic rigor. Discrepancies were discussed until consensus was reached. A senior researcher provided input to resolve disagreements when necessary. Throughout data collection and analysis, the researchers maintained a reflexive journal to record assumptions, expectations, and reactions arising from their clinical backgrounds and prior familiarity with the topic. Bracketing was used to help minimize the influence of preconceptions on interpretations.

### 2.5 Rigor and trustworthiness

Several strategies were used to enhance rigor and trustworthiness. Credibility was supported through prolonged engagement with the data, repeated reading of transcripts, use of field notes, and participant resonance checking with a subset of participants. Dependability was strengthened by maintaining an audit trail of coding decisions, theme refinement, and consensus discussions. Confirmability was supported through reflexive journaling and team-based interpretation. Transferability was enhanced by providing a detailed description of the sample, setting, and analytic procedures.

### 2.6 Ethical considerations

The study was approved by the Ethics Committee of Zhejiang University School of Medicine (Reference number: 20240633). The study was conducted in accordance with the Declaration of Helsinki. Written informed consent was obtained from all participants before data collection. Participants were informed that they could decline or withdraw at any time without affecting their care. All identifying information was removed from transcripts, and participants were assigned codes (P1–P12) to protect confidentiality.

## 3 Results

### 3.1 Participant characteristics

Twelve women with gynecologic malignancies participated in the interviews. Their ages ranged from 30 to 79 years. Most participants had ovarian cancer (66.7%), and most were within the first year after diagnosis. The sample included women with varied educational backgrounds, marital statuses, and residence types. Interview duration ranged from 25 to 47 min. Participant characteristics are shown in Table 1.

**Table 1:** Participant characteristics (*N* = 12).

Participant	Age (years)	Diagnosis	Education level	Marital status	Residence	Occupation	Number of children	Time since diagnosis (months)	Treatment
P1	44	Ovarian cancer	Bachelor’s degree	Married	Urban	Company employee	2	4	Surgery
P2	69	Ovarian cancer	Primary school	Widowed	Rural	Farmer	2	5	Surgery
P3	74	Cervical cancer	Junior secondary	Married	Urban	Retired	1	24	Chemotherapy
P4	79	Ovarian cancer	Junior secondary	Widowed	Rural	Housewife	1	3	Chemotherapy
P5	52	Ovarian cancer	Junior secondary	Married	Urban	Retired	1	18	Chemotherapy
P6	64	Cervical cancer	Primary school	Married	Rural	Retired	1	6	Surgery
P7	47	Cervical cancer	Senior secondary	Divorced	Urban	Housewife	2	2	Surgery
P8	54	Ovarian cancer	Primary school	Married	Rural	Farmer	2	48	Chemotherapy
P9	47	Ovarian cancer	Senior secondary	Married	Rural	Farmer	1	5	Surgery
P10	30	Ovarian cancer	College	Married	Urban	Company employee	0	2	Surgery
P11	75	Endometrial cancer	Primary school	Married	Rural	Farmer	3	8	Chemotherapy
P12	54	Ovarian cancer	Junior secondary	Married	Urban	Retired	1	17	Chemotherapy

Median age was 57 years (range, 30–79). Data are presented as individual participant characteristics to support transparency in sampling.

### 3.2 Themes identified from the interviews

**Figure 2:**
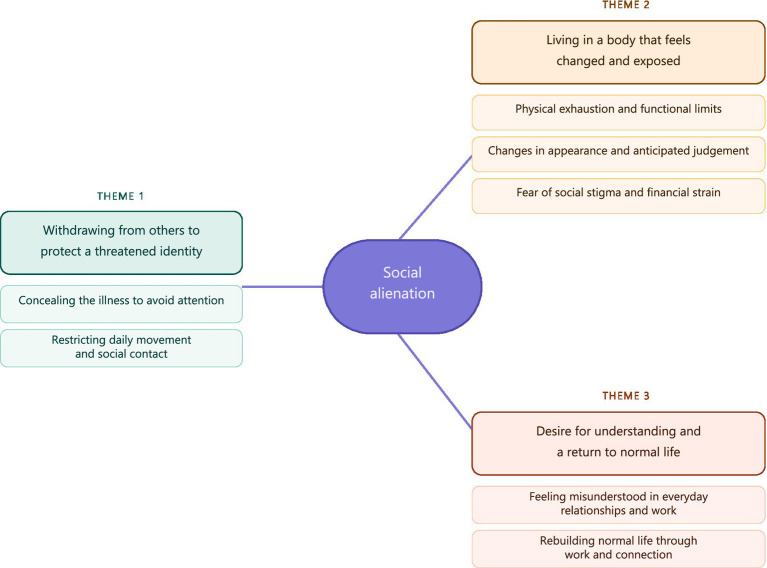
Thematic structure of social alienation among Chinese women with gynecologic malignancies.

Analysis of the interview transcripts followed Colaizzi’s seven-step method. The researchers identified significant statements after repeatedly reading the transcripts. These statements reflected efforts to conceal the illness and avoid public attention. Participants also described restricted daily activities, physical exhaustion, and embarrassment related to changes in appearance. Concerns about family reputation and financial burden were also reported. Many women expressed a desire to return to work and resume normal social participation. These statements were coded, compared across participants, and clustered through team discussion and consensus. This process yielded three themes and eight subthemes. The thematic structure is presented in Figure 2, and the subthemes are summarized in Table 2. The application of Colaizzi’s seven-step phenomenological analysis in this study is summarized in Table 3.

**Table 2:** Themes, subthemes, and representative quotations.

Theme	Subtheme	Representative quotation
Withdrawing from others to protect a threatened identity	Concealing the illness to avoid attention	*“I used to have many friends… but now, when I walk, I consciously choose paths where there are no people.” (P2)*
		*“I now have to walk by myself. When I go home, I do not want to be seen… I’m afraid of what people might say.” (P2)*
	Restricting daily movement and social contact	*“I do not participate in anything anymore. I’ve simplified everything.” (P3)*
		*“I just stay at home, alternating between the bed and the sofa.” (P4)*
		*“I have not even been downstairs… the furthest I go is the balcony.” (P10)*
Living in a body that feels changed and exposed	Physical exhaustion and functional limits	*“The numbness in my feet is severe, and I cannot even walk properly.” (P4)*
		*“I used to enjoy going out with friends… but now I cannot handle it; I just feel drained and tired.” (P5)*
		*“Because my health is poor, my constitution is weak. If I go out, I’m afraid of getting an infection.” (P7)*
	Changes in appearance and anticipated judgment	*“I just wish my hair would not fall out.” (P2)*
		*“I care about my appearance. I do not want people to see me without hair and laugh at me.” (P4)*
		*“I worry about this catheter. How are you supposed to socialise with a catheter?” (P7)*
	Fear of social stigma and financial strain	*“I’m worried potential partners [for my child] will say, ‘Her mother had such an illness.’ I’m worried it will affect my child’s future life.” (P7)*
		*“I rarely go out now because every trip costs money… I try to save wherever possible.” (P1)*
		*“Wigs are just too expensive, and I feel too embarrassed to see people like this [without a wig].” (P4)*
Desire for understanding and a return to normal life	Feeling misunderstood in everyday relationships and work	*“Some people, once they know about this disease, keep their distance… I do not want to associate with that kind of person either.” (P1)*
		*“My supervisor advised me not to come to work. They were worried I might catch a cold… but they were also afraid of potential trouble.” (P1)*
		*“No one else can understand these feelings. It’s only here [in the hospital] that I can talk easily… There’s a mutual understanding.” (P9)*
	Rebuilding normal life through work and connection	*“Having something to do would make me feel better. Otherwise, being idle makes me feel completely worthless.” (P1)*
		*“I really want to work now. Having a purpose would be very good for me.” (P9)*
		*“I still want to get back to how things were before, being able to meet friends and chat, not always hiding away.” (P7)*
		*“Do you have psychological counselling services here? I feel I really need it. You should really establish one.” (P10)*

P = participant code. Quotations were translated from Mandarin Chinese into English for publication.

**Table 3:** Colaizzi’s seven-step phenomenological analysis used in this study.

Colaizzi step	Analytic action in this study
Step 1: reading and familiarization	The researchers repeatedly read the interview transcripts and reviewed field notes to become immersed in the data.
Step 2: extracting significant statements	Statements related to concealment, withdrawal, bodily change, stigma, family concern, work, and reconnection were identified across all transcripts.
Step 3: formulating meanings	Meanings were developed from participants’ descriptions, such as social withdrawal to avoid attention, physical exhaustion limiting participation, and longing for understanding and ordinary life.
Step 4: clustering meanings into subthemes	Related meanings were grouped into subthemes and then compared across participants during team discussion.
Step 5: developing an exhaustive description	The researchers synthesized the subthemes into a comprehensive description of the social alienation experience.
Step 6: identifying the fundamental structure	The essential structure of the phenomenon was articulated as a multidimensional experience involving withdrawal, bodily exposure, perceived judgment, and reconnection needs.
Step 7: verification by participants	The preliminary synthesis was returned to three participants for member checking, and no major revisions were required.

This table describes the analytic procedure used to derive the themes. If the journal space is tight, this table can be moved to Supplementary material or the Methods section.

### 3.3 Theme 1: Withdrawing from others to protect a threatened identity

Participants often described reducing contact with others and limiting their visibility in public. This withdrawal was presented as a way of managing discomfort, avoiding questions, and protecting themselves from unwanted attention.

#### 3.3.1 Concealing the illness to avoid attention

Several women said they tried not to draw attention to their illness in daily life. Some avoided crowded places, changed the routes they took when walking, or preferred to remain unseen by others. One participant said, *“I used to have many friends… but now, when I walk, I consciously choose paths where there are no people”* (P2). Another explained, *“I now have to walk by myself. When I go home, I do not want to be seen… I’m afraid of what people might say”* (P2). These accounts show that concealing illness was often described as a practical response to anticipated discomfort in social settings, refer to Table 2.

#### 3.3.2 Restricting daily movement and social contact

Many participants also described a broader reduction in social activity. They no longer joined leisure activities, visited friends, or participated in community life as they had before diagnosis. One woman said, *“I do not participate in anything anymore. I’ve simplified everything”* (P3). Another stated, *“I just stay at home, alternating between the bed and the sofa”* (P4). A participant added, *“I have not even been downstairs… the furthest I go is the balcony”* (P10). Some women considered this narrowing of movement was accompanied by reduced contact with friends and colleagues and a loss of former daily routines, refer to Table 2.

### 3.4 Theme 2: Living in a body that feels changed and exposed

Participants described their bodies as a source of limitation, discomfort, and social vulnerability. Physical symptoms, appearance changes, and financial strain all contributed to reduced social participation.

#### 3.4.1 Physical exhaustion and functional limits

Treatment-related fatigue, numbness, and weakness limited participants’ ability to move freely and engage socially. One participant said, *“The numbness in my feet is severe, and I cannot even walk properly”* (P4). Another explained, *“I used to enjoy going out with friends… but now I cannot handle it; I just feel drained and tired”* (P5). A third participant stated, *“Because my health is poor, my constitution is weak. If I go out, I’m afraid of getting an infection”* (P7) These descriptions show how physical symptoms directly affected participants’ everyday mobility and willingness to go out.

#### 3.4.2 Changes in appearance and anticipated judgment

Many participants referred to hair loss, altered appearance, or medical devices as visible reminders of illness. These changes were often associated with embarrassment or concern about how others might respond. One participant said, *“I just wish my hair would not fall out”* (P2). Another stated, “*I care about my appearance. I do not want people to see me without hair and laugh at me”* (P4). A participant who had a catheter said, *“I worry about this catheter. How are you supposed to socialize with a catheter?”* (P7). These accounts indicate that visible bodily changes shaped how participants viewed themselves in social situations.

#### 3.4.3 Fear of social stigma and financial strain

Some participants worried that their diagnosis might affect their children’s future or bring unwanted attention to their family. One woman said, *“I’m worried potential partners [for my child] will say, ‘Her mother had such an illness.’ I’m worried it will affect my child’s future life”* (P7). Financial pressure was also a recurring concern. Several participants linked reduced social activity to treatment costs, loss of income, or the need to save money. One participant stated, *“I rarely go out now because every trip cost money… I try to save wherever possible”* (P1). Another said, “*Wigs are just too expensive, and I feel too embarrassed to see people like this [without a wig]”* (P4). For these women, financial strain and social withdrawal often appeared together, refer to Table 2.

### 3.5 Theme 3: Desire for understanding and a return to normal life

Although participants described withdrawal from others, they also expressed a strong wish to be understood and to re-engage with ordinary life.

#### 3.5.1 Feeling misunderstood in everyday relationships and work

Participants often felt that people around them did not fully understand what they were going through. Some described distance in friendships or family relationships, while others referred to work-related responses that they experienced as limiting. One participant said, *“Some people, once they know about this disease, keep their distance… I do not want to associate with that kind of person either”* (P1). Another described a workplace response: *“My supervisor advised me not to come to work. They were worried I might catch a cold… but they were also afraid of potential trouble”* (P1). A participant whose husband had died said, *“I’m just enduring each day now. If it were not for my child insisting on treatment, I would have followed my husband long ago”* (P4). Several participants said that fellow patients were among the few people who truly understood their experience. One woman explained, “*No one else can understand these feelings. It’s only here [in the hospital] that I can talk easily… There’s a mutual understanding”* (P9).

#### 3.5.2 Rebuilding normal life through work and connection

Despite their difficulties, many participants expressed a desire to resume work, return to normal routines, and reconnect with others. One participant said, *“Having something to do would make me feel better. Otherwise, being idle makes me feel completely worthless”* (P1). Another stated, *“I really want to work now. Having a purpose would be very good for me”* (P9). A participant added, *“I still want to get back to how things were before, being able to meet friends and chat, not always hiding away”* (P7). Some participants also asked for psychological support. One said, *“Do you have psychological counselling services here? I feel I really need it. You should really establish one”* (P10). These accounts suggest that participants wanted not only symptom relief, but also conditions that would allow them to reconnect socially and regain a sense of ordinary life. Across the three themes, several contextual considerations appeared to shape participants’ experiences of social alienation, including family concerns, workplace responses, public visibility, body image changes, financial pressure, and peer understanding. These contextual considerations are summarized in Table 4.

**Table 4:** Contextual considerations related to social alienation.

Contextual factor	Participant accounts	Analytic interpretation
Family concerns	Some participants worried that the diagnosis might affect children’s future marriage or social standing.	Family-related concerns may intensify withdrawal for some women, particularly when illness is perceived as affecting relatives as well as the patient.
Workplace responses	Some participants described advice not to return to work as protective, but also limiting.	Workplace responses may be experienced as supportive, excluding, or both, depending on how they are communicated and received.
Public visibility	Participants often avoided crowded settings and preferred not to be noticed.	Anticipated judgment and loss of privacy may contribute to social withdrawal.
Body image changes	Hair loss, catheters, and fatigue were described as socially difficult.	Visible treatment effects can increase self-consciousness and reduce confidence in social participation.
Financial pressure	Participants linked reduced outings and appearance management to cost.	Economic strain can reinforce social withdrawal by restricting both participation and coping options.
Peer understanding	Several participants said only fellow patients truly understood them.	Peer support may reduce alienation by providing recognition and shared experience.

This table presents the authors’ interpretive synthesis of participant accounts and should be read as a contextual summary rather than a claim of universal causation.

## 4 Discussion

### 4.1 Principal findings

This phenomenological study explored the experiences of social alienation among Chinese women with gynecologic malignancies. The findings suggest that social alienation in this context is not limited to reduced social contact. It involves a broader experience of withdrawal, felt estrangement, bodily exposure, and difficulty maintaining ordinary social roles. Participants described avoiding attention, limiting movement and interaction. Participants described feeling embarrassed by bodily changes, worrying about family reputation and financial strain. They also showed a desire to be understood and to return to ordinary life. The accounts suggest that social alienation emerged from the interaction of symptom burden, body-related distress, perceived judgment, social misunderstanding, and practical constraints. Although participants often withdrew from social contact, their narratives consistently reflected a strong desire for reconnection, indicating that alienation was experienced as an externally imposed condition rather than a voluntary state.

### 4.2 Social withdrawal in response to anticipated judgment

In this sample, many participants described deliberately reducing visibility in public spaces. This observed pattern in the present study may reflect more than simple social avoidance. In the present study, withdrawal often appeared to function as a protective response to anticipated attention, questions, or discomfort. This finding is consistent with prior research showing that cancer-related stigma, fear of negative evaluation, and concern about being treated differently can shape social participation (6, 12, 17). These concerns may be intensified by the visible effects of treatment among women with gynecologic malignancies.

Among the participants in this study, the perceived sensitivity surrounding reproductive health conditions appeared to further compound these experiences. Difficulties in openly discussing such issues may also contribute to the persistence and escalation of these concerns. However, these meanings should not be considered universal. The data indicate that, for some participants, social withdrawal functioned as a practical coping strategy to preserve dignity and maintain emotional safety in everyday life. Some accounts suggested that women interpreted others’ responses as distancing or unsupportive, which may have reinforced withdrawal. In this sense, alienation appears to be relational as well as individual. It is shaped not only by the woman’s own reactions, but also by the anticipated and actual responses of people around her (8, 16).

### 4.3 Embodiment, appearance, and role disruption

A central feature of the participants’ accounts was the sense that the body had become changed and exposed that made difficult to manage socially. Fatigue, numbness, weakness, hair loss, and medical devices were not only considered as physical symptoms. They were also experienced as social burdens that affected patients’ confidence and visibility during interaction. This is consistent with qualitative studies in cancer survivorship showing that body changes can alter how women feel about themselves in public and private life (24). In the present study, appearance-related concerns were closely linked to anticipated judgment and embarrassment. Some women worried that others would stare, laugh, or gossip about their appearance. Others avoided social situations because they did not want their bodily changes to be seen.

Participants also linked illness to changes in family and social roles. In the present study, several participants expressed concern that their diagnosis might affect family members. These accounts not necessarily indicate a fixed cultural pattern but they also suggest that family reputation and intergenerational consequences may be meaningful considerations for some women in this context (6, 23). Such concerns may deepen alienation by making illness feel socially consequential beyond the individual patient.

Financial pressure was another important part of the experience. Treatment costs, reduced income, and the expense of managing appearance changes, such as wigs also contributed to social withdrawal. This finding is consistent with broader research on financial toxicity in cancer care. Previous studies have shown that economic burden affects not only access to treatment, but also personal identity, social participation, and emotional wellbeing (8).

### 4.4 Social disconnection and the desire for reconnection

Participants often withdrew from social interactions but did not perceive this withdrawal as desirable. Many expressed a strong desire to be understood, regain a sense of purpose, and return to ordinary social life. These experiences were particularly evident in narratives related to employment and peer support also the wish to stop concealing their condition. Some participants perceived workplace responses as outwardly supportive but also restrictive. Advice against returning to work was often presented as a protective measure (25). However, several women interpreted these responses as exclusionary and as an indication that they continued to be viewed as vulnerable or incapable (26–28). These experiences may reflect a form of well-intentioned but limiting support. This could be cautiously interpreted as benevolent exclusion. However, this should be presented as an interpretive possibility rather than a definitive structural mechanism.

Work emerged as an important aspect of recovery and social reintegration. Several women considered employment represented more than financial stability. It also provided structure, purpose, and a sense of self-worth. Their desire to return to work suggests that reintegration not only involves restoring social contact but also reclaiming identity and participation in everyday life (29). Participants also identified fellow patients as a particularly meaningful source of support. Shared experiences appeared to foster understanding and recognition that were not always available within family or workplace environments (30, 31). These findings highlight the potential value of peer-based and relational support that is both emotionally responsive and grounded in lived experience.

### 4.5 Implications for practice

The findings of this study have several implications for clinical practice. Social alienation should be considered during the psychosocial assessment of women with gynecologic malignancies. It is particularly important among patients reporting avoidance behaviors, shame, diminished self-confidence, or withdrawal from occupational and social activities. Assessment should extend beyond emotional distress to include body image concerns, communication challenges, and disruption of social and familial roles (32).

Family-oriented support approaches may be beneficial for women who feel misunderstood or who are concerned about the impact of their illness on family members. Educational interventions for relatives should emphasize empathetic listening, emotional validation, and practical assistance. Such approaches may be more supportive than protective behaviors that unintentionally limit patient autonomy or decision-making (32–35). Peer support may also represent an important supportive resource (27, 35–37). Participants frequently described fellow patients as easier to communicate with and more capable of understanding their experiences. Shared lived experience appeared to foster recognition, acceptance, and emotional connection. These factors may help reduce feelings of isolation among them.

The importance participants placed on employment and everyday normality suggests that rehabilitation and return-to-work support may be relevant for selected patients. These interventions should be individualized and adapted to treatment stage, physical functioning, and patient preferences. Digital support platforms may also provide an additional avenue for support (24, 36–38). It is particularly important for women who avoid face-to-face interaction. However, this possibility should be interpreted cautiously, as digital interventions were not directly examined in the present study.

Future supportive interventions should remain culturally responsive and avoid assumptions that all women share similar family expectations or social concerns (33, 35–39). The findings indicate that social alienation is shaped by the interaction between illness-related experiences, embodied changes, and social responses. Accordingly, supportive care should be flexible, individualized, and patient-centered.

### 4.6 Strengths and limitations

A major strength of this study is its exploration of social alienation from the lived perspectives of women with gynecologic malignancies using a phenomenological approach. The in-depth interviews generated rich narrative data that enabled a nuanced understanding of experiences that may not be adequately captured through quantitative methods. The findings highlighted the interrelated nature of social withdrawal, bodily exposure, family-related concerns, and the desire for social reconnection.

Several limitations should also be acknowledged. First, the study was conducted in a single tertiary hospital in Hangzhou, which may limit the transferability of the findings to other geographic regions or healthcare settings. In addition, recruitment from a tertiary hospital may have introduced selection bias. As patients in this setting are likely to differ from those receiving care in community hospitals or rural healthcare facilities, they may differ in education level, socioeconomic status, and access to psychosocial support. Findings may therefore not be fully transferable to populations with limited access to tertiary care.

Second, the sample size was appropriate for phenomenological inquiry. However, the number of participants was relatively small. Third, the cross-sectional design captured experiences at a single point in the illness trajectory and therefore could not examine changes over time. The findings were derived exclusively from patient self-report and were not triangulated with perspectives from caregivers, healthcare professionals, or employers. Translation of interview data from Chinese into English may also have resulted in some loss of linguistic or cultural nuance within selected quotations. Finally, member checking was conducted with three of the 12 participants. The perspective of the remaining nine participants was not directly verified, which may represent a limitation of the confirmability strategy.

## 5 Conclusion

Chinese women with gynecologic malignancies described social alienation as a multidimensional experience. It included social withdrawal, bodily vulnerability, perceived judgment, financial strain, and a persistent desire for understanding and reconnection. The findings suggest that social alienation is not limited to reduced social participation. It also reflects a subjective sense of disconnection shaped by illness-related experiences, embodied change, and interpersonal relationships. Supportive care should therefore address both practical and relational needs, with attention to family communication, peer support, and opportunities for meaningful social re-engagement.

## Data Availability

The raw data supporting the conclusions of this article will be made available by the authors, without undue reservation.
